# Extracellular vesicle-derived DNA for performing *EGFR* genotyping of NSCLC patients

**DOI:** 10.1186/s12943-018-0772-6

**Published:** 2018-01-27

**Authors:** Jae Young Hur, Hee Joung Kim, Jong Sik Lee, Chang-Min Choi, Jae Cheol Lee, Min Kyo Jung, Chan Gi Pack, Kye Young Lee

**Affiliations:** 1 Lung Cancer Center, Konkuk University Medical Center, Seoul, Republic of Korea; 2 Department of Pathology, Konkuk University Medical Center, Seoul, Republic of Korea; 3 Department of Pulmonary Medicine, Konkuk University School of Medicine, 120-1 Hwayang-dong, Gwangjin-Gu, Seoul, 05030 Republic of Korea; 4 Department of Pulmonary and Critical Care Medicine, University of Ulsan, College of Medicine, Asan Medical Center, Seoul, Republic of Korea; 5 Department of Oncology, University of Ulsan, College of Medicine, Asan Medical Center, Seoul, Republic of Korea; 6 Department of Convergence Medicine, University of Ulsan, College of Medicine & Asan Institute for Life Sciences, Asan Medical Center, Seoul, Republic of Korea

**Keywords:** Liquid biopsy, Bronchoalveolar lavage fluid, Extracellular vesicles, *EGFR* mutant DNA, Non-small cell lung cancer

## Abstract

Tumor cells shed an abundance of extracellular vesicles (EVs) to body fluids containing bioactive molecules including DNA, RNA, and protein. Investigations in the field of tumor-derived EVs open a new horizon in understanding cancer biology and its potential as cancer biomarkers as well as platforms for personalized medicine. This study demonstrates that successfully isolated EVs from plasma and bronchoalveolar lavage fluid (BALF) of non-small cell lung cancer (NSCLC) patients contain DNA that can be used for *EGFR* genotyping through liquid biopsy. In both plasma and BALF samples, liquid biopsy results using EV DNA show higher accordance with conventional tissue biopsy compared to the liquid biopsy of cfDNA. Especially, liquid biopsy with BALF EV DNA is tissue-specific and extremely sensitive compared to using cfDNA. Furthermore, use of BALF EV DNA also demonstrates higher efficiency in comparison to tissue rebiopsy for detecting p.T790 M mutation in the patients who developed resistance to EGFR-TKIs. These finding demonstrate possibility of liquid biopsy using EV DNA potentially replacing the current diagnostic methods for more accurate, cheaper, and faster results.

**Electronic supplementary material:**

The online version of this article (10.1186/s12943-018-0772-6) contains supplementary material, which is available to authorized users.

## Main text

Lung cancer results in the largest number of cancer-related deaths worldwide and non-small-cell lung cancer (NSCLC) accounts for more than 85% of all lung cancer cases [1]. Most patients are diagnosed at an advanced stage due to lack of efficient diagnostic approaches and asymptomatic characteristic of the disease leading to a poor prognosis [1, 2]. Recent development of target specific drugs such as epidermal growth factor receptor-tyrosine kinase inhibitors (EGFR-TKIs) have slightly improved survival rate, but easy and fast diagnostic assessment of mutation status is important for timely treatment of patients. At present, majority of *EGFR* genotyping is done through tissue biopsy while liquid biopsies using cell-free DNA (cfDNA) are used as supplement tests [3, 4]. The conventional tumor biopsy to assess mutation status can be problematic depending on the location and size of the tumor. Liquid biopsy, a noninvasive way to detect circulating tumor DNA (ctDNA) in the blood, are proposed as an alternative way to detect, evaluate and monitor tumor-drug relation [3, 5]. The integration of liquid biopsy into cancer treatment depends on the precision of detecting ctDNA in blood samples, but plasma cfDNA only contains roughly 1% of ctDNA [6]. Therefore, even with high specificity reported in using ctDNA, varied sensitivity is a problem. For example, some studied reported relatively high sensitivities ranging from 66% to 78%, while other studies resulted low sensitivities ranging from 28.8% to 46%. [7–10].

The main reason for this high variability of sensitivity ctDNA lies on the unstable nature of cfDNA in the samples [7]. In contrast, DNA inside extracellular vesicle (EV) shed by tumor cells is well protected by dual lipid membranous coating and thus has inherent stability [5, 11, 12]. Along with abundant new discoveries in various tumor-derived EVs and EV-derived DNA (EV DNA), they have great potential as cancer biomarkers as well as platforms for personalized medicine [12–14]. For example, Thakur BK, et al. have demonstrated that the majority of DNA associated with tumor exosomes is double-stranded in various cancer cell-lines and highlight the translational value of exosomal DNA for its potential usefulness as a circulating biomarker for cancer detection [12].

## Visualization and characterization of EVs isolated from the BALF and plasma NSCLC patients

**Fig. 1:**
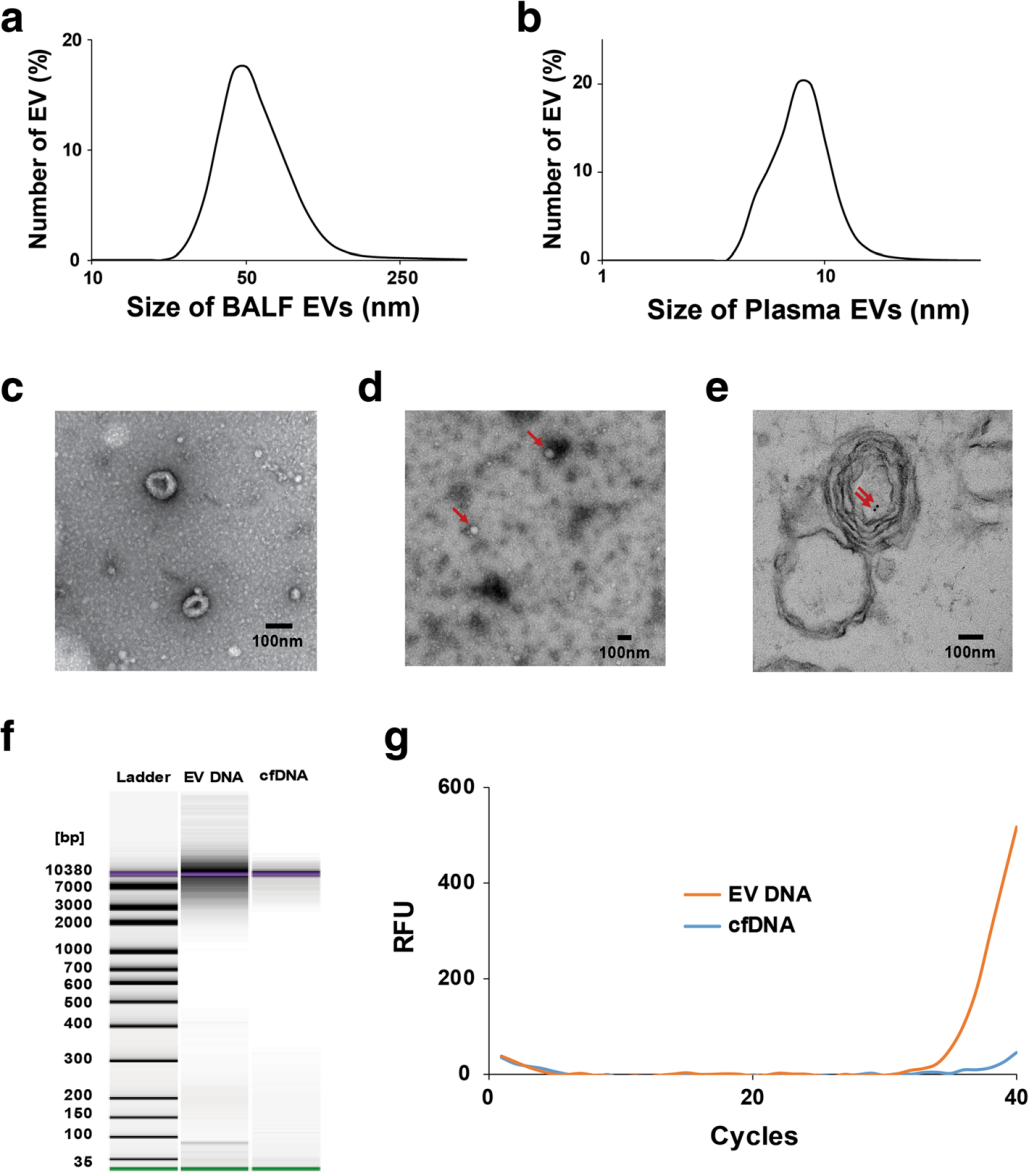
Characterization of BALF and plasma EV DNA. **a.** Size distribution of BALF EV. BALF was ultracentrifuged to obtain pallets and remove cells and debris, which was resuspended in 200 μl PBS. Sizes of purified EVs were determined using Zetasizer Nano ZS. Average size distribution from six separate experiments is plotted in percentage distribution according to their size. All six distributions are shown in Additional file 1: Figure S1. **b.** Size distribution of plasma EV. Plasma was ultracentrifuged to obtain pallets and remove cells and debris, which was resuspended in 200 μl PBS. Sizes of purified EVs were determined using Zetasizer Nano ZS. Average size distribution from three separate experiments is plotted in percentage distribution according to their size. All three distributions are shown in Additional file 1: Figure S2. **c.** EM image of BALF EVs. Samples for EM analysis were negatively stained. The size bar in the EM image indicates 100 nm. **d.** EM image of plssma EVs. Samples for EM analysis were negatively stained. Plasma EVs indicated by red arrows. The size bar in the EM image indicates 100 nm. **e.** Detection of dsDNA in BALF EVs by performing immuno-EM. DsDNA was labeled with a mouse monoclonal antibody and colloidal gold-conjugated secondary antibodies. The solid black dots indicate DNA (indicated by red arrows). **f.** Gel-like images show the size and amount of EV DNA and cfDNA determined using the bioanalyzer. First lane shows the standard size ladder distribution, and numbers on the left indicate corresponding sizes. The second and third lanes show the size and amount of EV DNA and cfDNA, respectively. **g.** Amplification curve obtained by performing real-time PCR. Exon 19 deletion in *EGFR* was determined by performing peptide nucleic acid (PNA)-mediated PCR clamping. Both EV DNA and cfDNA were extracted from 1 ml BALF, and 70 ng EV DNA and cfDNA were used for performing PCR

Previous studies report that measurements of EV size varies according to the methods of the isolation and measurement, but EVs from human body fluids (plasma, urine) are generally found to be in 20~ 300 nm range [15–17]. This study identified the size of BALF EVs to be from 20 to 250 nm (Fig. 1a and Additional file 1: Figure S1) and the size of plasma EV to be smaller ranging from 5 to 15 nm (Fig. 1b and Additional file 1: Figure S2). Visualization of purified EV fraction showed round shape and heterogeneous in size, averaging in 106 nm (SD ± 34 nm) BALF EVs (Fig. 1c and Additional file 1: Figure S3). Image obtained from EM of plasma EVs showed other substances in the background, which are probably proteins, low-density lipoproteins (LDL), and high-density lipoproteins (HDL) that exist in plasma (Fig. 1d).

Results of immuno-electron microscopy (immune-EM) identified the presence of dsDNA in the intraluminal portion of EVs covered by a multilayered or single-layered membrane (Fig. 1e and Additional file 1: Figure S4). NanoDrop analysis showed that both EV DNA and cfDNA obtained from BALF had similar concentration and purity (Additional file 1: Table S2). Bioanalyzer analysis of dsDNA length showed that the size of both EV DNA and ctDNA are > 3 kb, but abundance of dsDNAs that are longer than 3 kb is greater in EV DNA compared to ctDNA (Fig. 1f, Additional file 1: Table S3 and Figure S5). We compared the sensitivity of *EGFR* mutation testing between BALF EV DNA and cfDNA by performing PCR. Results showed higher sensitivity of *EGFR* mutation testing when using EV DNA compared to using cfDNA. For example, a threshold cycle (Ct) value of EV DNA was 32, whereas that of cfDNA could not be determined (Fig. 1g and Additional file 1: Table S4).

## Liquid biopsy of plasma EV DNA resulted in higher accordance rate compared to liquid biopsy of plasma cfDNA

At present, blood plasma DNA samples are widely used for liquid biopsies and the major concern is the instability of the DNA from the blood sample, which leads to decreased sensitivity. Presumably, using plasma EV DNA instead of cfDNA could increase sensitivity as they are shielded from the outer environment by lipid bilayer structure of EV. We performed *EGFR* genotyping with 20 plasma samples of NSCLC patients. Comparison of the results with tissue biopsy results showed that plasma cfDNA only had 30% accordance with tissue typing. Even though detection sensitivity with plasma cfDNA was relatively low, we were able to improve detection sensitivity to 55% by using plasma EV DNA (Additional file 1: Table S5). This finding suggests that liquid biopsy using EV DNA is advantageous to conventional use of cfDNA. Recently, Allenson et al. reported that liquid biopsy of exo-DNA was superior to that of cfDNA for detecting mutant *KRAS* in plasma samples of patients with pancreatic ductal adenocarcinoma [11]. However, isolation and purification of EVs from plasma is associated with some technical difficulties. Characteristics of lipoproteins, especially low-density lipoproteins (LDLs), present in the plasma are very similar to those of EVs; moreover, contamination of isolated EVs samples with LDLs interferes with analysis [18]. Components similar to LDLs were identified in our study after isolating EVs from plasma (Fig. 1d), which could explain the relatively low sensitivity of liquid biopsy when using plasma EV DNA.

## Liquid biopsy using BALF EV DNA for *EGFR* mutation testing is sendant with tissue genotyping

Although, the standard for non-invasive cancer diagnostics is detection of biomarkers circulating in blood, it remains a challenge due to abundant non-cellular contents. Our approach to overcome this problem is to analyze more immediate biofluids such as bronchoalveolar lavage fluid (BALF) of NSCLC patients. Proximal biofluids display component specificity and in some cases, they are in direct contact with the site of the disease. Bronchoalveolar washing is not an entirely non-invasive procedure, but often ordered in individuals with and suspected of lung cancer during bronchoscopy.

Higher quantity and quality of EV DNA compared to BALF cfDNA suggested a potential for clinical application of EV DNA for liquid biopsy. Best way to test the specificity and sensitivity of liquid biopsy done with BALF EV DNA is comparing the results with conventional tissue biopsy. Therefore, we tested 23 BALF samples from NSCLC patients with proven *EGFR* genotyping from tissue biopsy (9 *EGFR* wild type and 14 *EGFR*-mutated). *EGFR* genotyping using BALF EV DNA showed 100% accordance with tissue typing, while detection sensitivity using ctDNA only yielded 71.4% (Table 1). Test results of BALF samples were significantly higher compared to the result of plasma samples in both ctDNA and EV DNA, demonstrating that proximal biofluids better represents tumor status. In addition, as we have seen with the plasma sample, EV DNA resulted in higher specificity and sensitivity. Specifically, Kappa coefficient for *EGFR* genotyping by using tumor tissue sample and BALF EV DNA was 1.0 (*p* < 0.01), which was higher than that for *EGFR* genotyping by using tumor tissue sample and BALF ctDNA (kappa = 0.705). Our results of liquid biopsy using BALF EV DNA was compatible enough with tissue genotyping that it can replace tissue biopsy when obtaining tissue sample is difficult. Additionally, liquid biopsy could significantly reduce turn-around time, which usually takes two or three weeks for *EGFR* genotyping using tissue biopsy.

**Table 1:** Comparison of the EGFR mutation status between tumor tissue and BALF in EGFR-TKIs naïve patients

EGFR genotype	Tissue	BALF (*n* = 23)			
		EV DNA		cfDNA	
		Mutant type	Wild type	Mutant type	Wild type
Mutant type	14 (60.9%)	14 (60.9%)	0	10 (43.5%)	0
Wild type	9 (39.1%)	0	9 (39.1%)	4 (17.4%)	9 (39.1%)
Sensitivity(%)(95% CI)		100.0% (85.7–100)		71.4% (51.0–85.7)	
Specificity (%) (95% CI)		100.0% (85.7–100)		100.0% (85.7–100)	

*Abbreviations*: *CI* Confidence interva

## Genotyping using BALF EV DNA is highly promising for p.T790 M detection for acquired resistance patients

**Fig. 2:**
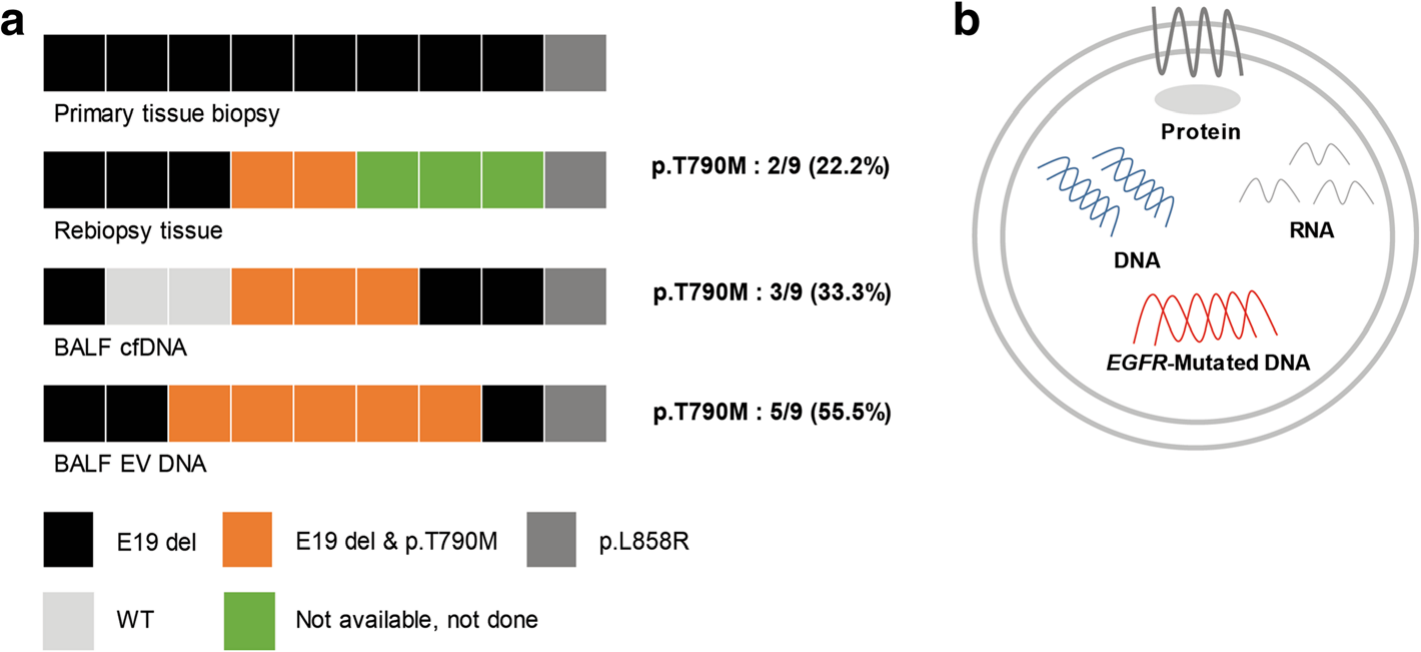
Detection of EGFR mutation by using BALF EV DNA. **a.** Comparison of EGFR genotyping results performed using tumor tissue, BALF cfDNA, and BALF EV DNA. The top lane indicates EGFR mutation status in the primary tumor tissue of each patient. The second lane shows the result of biopsy of tumor tissues obtained from patients with acquired resistance to EGFR-TKIs. The third and fourth lanes show EGFR mutation status determined by performing liquid biopsy of BALF cfDNA and EV DNA, respectively. Rates of p.T790 M mutation detection by performing tissue rebiopsy and liquid biopsy of BALF cfDNA and BALF EV DNA are shown on the right side of each lane. Abbreviations; E19 del: exon 19 deletion, WT: wild type. **b.** Diagram showing the contents of an EV. Tumor-derived EVs contain RNA; DNA, including mutant DNA; and proteins

Unfortunately, most patients prescribed with TKI after identification of mutations such as Exon 19 deletion and p.L858R acquire resistance to the drug after prolonged treatment. Hence, precise and swift identification of secondary mutations, p.T790 M, that represent acquired resistance is important for planning future treatment. We extended liquid biopsy using BALF to the patients who developed acquired resistance to EGFR-TKIs and need rebiopsy to further test specificity and sensitivity. Nine patients who developed resistance to TKIs were examined for the p.T790 M mutation by performing conventional tissue rebiopsy and liquid biopsy with BALF samples. Of these nine patients, suitable cancer cells to perform tissue rebiopsy were obtained from only six patients, while adequate tissues were unobtainable from three patients. Two patients out of six patients were identified to have the p.T790 M mutation from tissue rebiopsy (Fig. 2a and Additional file 1: Table S6).

When we performed liquid biopsy of BALF cfDNA three patients were identified to have p.T790 M mutation including two patients who yielded positive results from the tissue rebiopsy as well as one patient who were not identified by the tissue rebiopsy (Fig. 2a and Additional file 1: Table S6). Sensitivity of liquid biopsy increases even further when tested with BALF EV DNA with total of five patients identified to have p.T790 M mutation including two patients who yielded positive results from the tissue rebiopsy as well as three patients who were not identified by the tissue rebiopsy. Three additionally identified patients consists of two patients who could not provide adequate tissue sample for performing tissue rebiopsy and one patient who yielded negative result for p.T790 M mutation from tissue rebiopsy (Fig. 2a and Additional file 1: Table S6). Furthermore, two newly identified patients showed partial response to subsequent osimertinib treatment (Additional file 1: Table S6). We demonstrated for the first time that EVs isolated from BALF of NSCLC patients carry genomic dsDNA and specific mutant *EGFR* DNA inside the double layered membranous vesicles (Fig. 2b). Clinical usefulness of genotyping using BALF EV DNA was more prominent in the matter of detecting p.T790 M mutation for the prescription of the 3rd generation EGFR-TKIs such as osimertinib [19]. Although the present study did not include a large sample size, preliminary findings of this study suggest that liquid biopsy of BALF EV DNA can overcome limitations associated with tissue rebiopsy, which is widely performed for detecting p.T790 M mutation.

## Conclusions

Our results show that EGFR mutation detection in NSCLC patients is possible through EGFR genotyping of EVs present in plasma and BALF. Liquid biopsy of BALF EV DNA is non-invasive, simple and faster testing method that is also high in accuracy and even surpass detection sensitivity compared to tissue biopsy. Sensitivity is shown to be especially high in acquired resistance patients. This study revealed a novel liquid biopsy method of using EV DNA for EGFR genotyping. It has demonstrated potential to serve as a diagnostic and prognostic method in NSCLC patients.

## Additional file

Additional file 1: Materials and methods. **Table S1.** Patient demographics and clinical characteristics. **Table S2.** Concentration and purity (260/280) of BALF EV DNA and BALF cfDNA. **Table S3.** Concentration of DNA larger than 1kb in EV DNA and cfDNA. **Table S4.** Ct value of EV DNA and cfDNA samples and their differences. **Table S5.** Comparison of the EGFR mutation status between tumor tissue and plasma in EGFR-TKIs naïve patients. **Table S6.** Clinical characteristics of patients who developed acquired resistance to 1st or 2nd generation EGFR-TKIs and underwent rebiopsy. **Figure S1.** Sizes of purified BALF EVs. **Figure S2.** Sizes of purified plasma EVs. **Figure S3.** EM image of BALF EVs. **Figure S4.** Immuno-EM images show detection of dsDNA in BALF EVs. Red arrows indicate gold particles. **Figure S5.** Gel-like images show the size and amount of EV DNA and cfDNA determined using the bioanalyzer. (DOCX 900 kb)

## Abbreviations

BALF: Bronchoalveolar lavage fluid
cfDNA: Cell-free DNA
ctDNA: Circulating tumor DNA
DLS: Dynamic light scattering
dsDNA: Double-stranded DNA
EGFR-TKIs: Epidermal growth factor receptor-tyrosine kinase inhibitors
EV DNA: Extracellular vesicle-derived DNA
EV: Extracellular vesicle
HDL: High-density lipoproteins
LDL: Low-density lipoproteins
NSCLC: Non-small cell lung cancer
PNA: Peptide nucleic acid
TEM: Transmission electron microscopy
