# Supplementary material for: Extracellular vesicle-derived DNA for performing EGFR genotyping of NSCLC patients
Source: Mol Cancer. 2018 Jan 27;17:15. doi: 10.1186/s12943-018-0772-6 (PMC5787306; doi:10.1186/s12943-018-0772-6)
Supplement: Additional file 1: — Materials and methods. Table S1. Patient demographics and clinical characteristics. Table S2. Concentration and purity (260/280) of BALF EV DNA and BALF cfDNA. Table S3. Concentration of DNA larger than 1kb in EV DNA and cfDNA. Table S4. Ct value of EV DNA and cfDNA samples and their differences. Table S5. Comparison of the EGFR mutation status between tumor tissue and plasma in EGFR-TKIs naïve patients. Table S6. Clinical characteristics of patients who developed acquired resistance to 1st or 2nd generation EGFR-TKIs and underwent rebiopsy. Figure S1. Sizes of purified BALF EVs. Figure S2. Sizes of purified plasma EVs. Figure S3. EM image of BALF EVs. Figure S4. Immuno-EM images show detection of dsDNA in BALF EVs. Red arrows indicate gold particles. Figure S5. Gel-like images show the size and amount of EV DNA and cfDNA determined using the bioanalyzer. (DOCX 900 kb) [file 12943_2018_772_MOESM1_ESM.docx]

**Additional files**

**Materials and methods**

**Patients and clinical samples**

Patients with advanced NSCLC were recruited according to a protocol approved by the institutional review board of Konkuk University Medical Center (KUMC) and Asan Medical Center (AMC), which requires written informed consent to be obtained from all patients. Plasma samples were obtained from 20 patients with *EGFR*-mutated advanced NSCLC who visited KUMC, and BALF samples were obtained from EGFR-TKI-naïve patients who visited AMC (20 patients) and KUMC (three patients). BALF samples for detecting p.T790M mutation were obtained from nine patients who visited KUMC (Table S1).

For obtaining plasma samples, 5 ml blood samples were collected from patients in K2 EDTA tubes and were centrifuged at 1000 × *g* and 4°C for 15 min. Supernatants obtained were stored at −80°C for EV isolation. BALF was collected in 50 ml centrifuge tubes and was immediately centrifuged at 1000 × *g* and 4°C for 15 min to remove cells and debris. Supernatants obtained were stored at −80°C for EV isolation.

**Isolation and characterization of EVs**

Plasma or BALF sample lacking cells and debris was transferred to an ultracentrifuge tube and was centrifuged at 200,000 × *g* and 4°C for 1 h by using a Beckman rotor (Beckman Coulter, Brea, CA, USA). EVs were isolated from 500 μl plasma and 1 ml BALF. Supernatant was carefully removed, and pellet was resuspended in 200 μl PBS. Sizes of purified EVs were analyzed using dynamic light scattering (DLS) technique with Zetasizer Nano ZS (Malvern Instruments, Worcestershire, UK) at a 90° fixed angle.

**Negative-stain transmission electron microscopy (TEM) and immuno-EM**

The EV fractions in BALF and plasma were visualized by negative stain transmission electron microscopy (TEM). For negative-stain TEM, purified EVs were fixed in 2% (vol/vol) paraformaldehyde for 5 min at room temperature. After fixation, 10 μl EV suspension was applied to formvar-/carbon-coated grids (200 mesh) for 1 min and was stained with 2% uranyl acetate. Excess uranyl formate was removed using a filter paper, and the grids were examined using a transmission electron microscope (H7600; Hitachi, Tokyo, Japan) at 80 kV.

For performing immuno-EM, EV pellets were fixed using 2.5% glutaraldehyde and 2% paraformaldehyde in sodium cacodylate buffer (pH 7.2) at 4°C. Next, the samples were fixed again by using 1% osmium tetra-oxide for 30 min at 4°C. The fixed samples were dehydrated using an ethanol series (50%, 60%, 70%, 80%, 90%, and 100% ethanol) for 20 min and were transferred to Spurr’s medium (Electron Microscopy Science, Hatfield, PA, USA). The samples were impregnated with and embedded into the same resin mixture, sectioned (60-nm-thick sections) with an ultramicrotome (Leica Ultracut UCT; Leica Microsystems, Vienna, Austria), and placed on nickel grids. DNA in the samples was labeled with immunogold by using a mouse monoclonal antibody (sc-58749; Santa Cruz Biotechnology, Dallas, TX, USA) and 9- to 11-nm colloidal gold-conjugated goat anti-mouse IgG secondary antibodies (Sigma, St. Louis, MO, USA). After immunogold labeling, the sections were double-stained with 2% uranyl acetate for 20 min and lead citrate for 10 min and were viewed under the transmission electron microscope.

**Elimination of free-floating DNA**

To eliminate free-floating DNA present outside EVs, purified EVs were placed in a microtube, treated with 10× reaction buffer (200 mM Tris-HCl [pH 8.3] and 20 mM MgCl_2_) and DNase I (Sigma, St. Louis, MO, USA), and incubated for 15 min at room temperature. To promote the binding of calcium and magnesium ions and to inactivate DNase I, the samples were treated with a stop solution (50 mM EDTA), heated at 70°C for 10 min, and chilled on ice.

**Extraction of EV DNA and cfDNA**

After eliminating free-floating DNA, EVs were lysed and EV DNA was purified using High-Pure PCR Template Preparation Kit (Roche Diagnostics, Mannheim, Germany). CfDNA present in cell-free BALF was also purified using High-Pure PCR Template Preparation Kit. The quality and length of the purified DNA were analyzed using a bioanalyzer and high-sensitivity DNA chip (Agilent Technologies, Santa Clara, CA, USA). The concentration and purity of the DNA samples were measured using NanoDrop machine (Thermo Scientific, Waltham, MA, USA).

***EGFR* mutation testing**

*EGFR* mutations were detected using PNAClamp^TM^ EGFR Mutation Detection Kit (Panagene, Daejeon, Korea) by performing peptide nucleic acid (PNA)-mediated PCR clamping. One sample was tested for 29 *EGFR* mutations in eight individual reaction tubes. All reaction tubes had 20 µl reaction mixture containing 70 ng template DNA, a primer, a PNA probe set, and PCR master mix containing a fluorescence dye. All reagents used were present in the kit. PNA-mediated PCR clamping was performed using CFX96^TM^ (Bio-Rad, Hercules, CA, USA). PCR conditions are as follows: initial denaturation at 94°C 5 min, followed by 40 cycles of 94°C for 30 s, 70°C for 20 s, 63°C for 30 s, and 72°C for 30 s. The efficiency of PCR clamping was determined by calculating Ct value. Ct values for control and mutation assays were calculated using fluorescence amplification plots. Delta Ct (ΔCt) value was calculated using the formula ΔCt-1 = standard Ct - sample Ct. Cut-off ΔCt-1 was defined as 2.0 for the 29 *EGFR* mutations [1].

**Statistical analysis**

Categorical variables were summarized by calculating frequencies and percentages. Means; standard deviations; and ranges, including minimal and maximal values, were used to determine numerical variables. Wilson score method was used to calculate 95% confidence intervals for concordance, sensitivity, and specificity. Concordance in the results of *EGFR* genotyping between tumor tissue and EV DNA or cfDNA samples were measured using Cohen’s kappa coefficient. Data were summarized as medians with interquartile range (IQR) with non-normal distribution. We used the Wilcoxon signed rank test to compare DNA concentration, purity and Ct values of EV and cf DNA. All statistical analyses were carried out using SPSS Statistics version 24 (IBM Corp, Chicago, IL, USA) and a P value < 0.05 was regarded as statistically significant.

**Reference**

1. Kim HJ, Lee KY, Kim YC, Kim KS, Lee SY, Jang TW, Lee MK, Shin KC, Lee GH, Lee JC, et al: Detection and comparison of peptide nucleic acid-mediated real-time polymerase chain reaction clamping and direct gene sequencing for epidermal growth factor receptor mutations in patients with non-small cell lung cancer. Lung Cancer 2012, 75:321-5.

**Table S1 Patient demographics and clinical characteristics**

| **Characteristic** | **N =52 (%)** |
| --- | --- |
| Age, yr |  |
| Median | 64.5 (56.3-71.0)* |
| Female | 36 (69.2%) |
| Stage at diagnosis |  |
| IB | 3 (5.8%) |
| IIA | 1 (1.9%) |
| IIIA | 2 (3.8%) |
| IIIB | 2 (3.8%) |
| IV | 44 (84.6%) |
| Histology |  |
| Adenocarcinoma | 50 (96.2%) |
| Squamous cell carcinoma | 2 (3.8%) |
| Tissue based EGFR genotyping |  |
| Exon 19 deletion | 25 (48.1%) |
| L858R | 17 (32.7%) |
| L858R, T790M | 1 (1.9%) |
| Wild type | 9 (17.%) |

*IQR= interquartile range

**Table S2 Concentration and purity (260/280) of BALF EV DNA and BALF cfDNA**

|  | **Concentration (ng/μl)** | | **260/280** | |
| --- | --- | --- | --- | --- |
|  | **EV DNA** | **cfDNA** | **EV DNA** | **cfDNA** |
| 1 | 13.7 | 15.2 | 1.3 | 1.5 |
| 2 | 13.3 | 11.1 | 1.8 | 1.8 |
| 3 | 10.9 | 10.0 | 1.4 | 1.6 |
| 4 | 15.0 | 15.0 | 1.4 | 1.5 |
| 5 | 20.6 | 22.2 | 2.1 | 1.7 |
| 6 | 13.7 | 14.5 | 1.7 | 1.4 |
| 7 | 10.4 | 8.5 | 1.6 | 2.0 |
| 8 | 12.4 | 12.0 | 1.6 | 1.7 |
| 9 | 10.1 | 9.0 | 1.6 | 1.5 |
| 10 | 12.1 | 13.8 | 1.5 | 1.6 |
| 11 | 10.8 | 13.8 | 1.5 | 1.4 |
| 12 | 9.6 | 12.9 | 2.1 | 1.6 |
| 13 | 9.1 | 11.3 | 1.7 | 1.5 |
| 14 | 19.3 | 12.0 | 1.6 | 1.7 |
| 15 | 11.2 | 11.2 | 1.6 | 1.4 |
| 16 | 29.4 | 15.5 | 1.3 | 1.3 |
| **Average** | 13.9 | 13.0 | 1.6 | 1.6 |
| **Median (IQR)** | 12.3 (10.5-14.7) | 12.5 (11.1-14.9) | 1.6 (1.4-1.7) | 1.6 (1.4-1.7) |
|  | P=1.0 | | P=0.568 | |

**Table S3 Concentration of DNA larger than 1kb in EV DNA and cfDNA.**

|  | **Concentration (pg/μl) of DNA >1kb** | |
| --- | --- | --- |
|  | **EV DNA** | **cf DNA** |
| 1 | 248.41 | 152.94 |
| 2 | 62.7 | 42.41 |
| 3 | 838.41 | 71.07 |
| 4 | 79.29 | 47.7 |
| 5 | 140.57 | 71.75 |
| **Median (IQR)** | 140.57 (71.00-543.41) | 71.07 (45.06-112.35) |
|  | P=0.043 | |

**Table S4 Ct value of EV DNA and cfDNA samples and their differences**

|  | **EV DNA (Ct)** | **cfDNA (Ct)** | **ΔCt (EV DNA (Ct) - cfDNA (Ct))** |
| --- | --- | --- | --- |
| 1 | 31.12 | not detected |  |
| 2 | 29.16 | 29.90 | -0.74 |
| 3 | 26.31 | 26.76 | -0.45 |
| 4 | 21.03 | 22.13 | -1.10 |
| 5 | 26.79 | 25.60 | 1.19 |
| 6 | 27.99 | 28.05 | -0.06 |
| 7 | 24.94 | not detected |  |
| 8 | 28.26 | not detected |  |
| 9 | 27.76 | 28.80 | -1.04 |
| 10 | 28.00 | 30.23 | -2.23 |
| 11 | 24.46 | 26.95 | -2.49 |
| 12 | 27.66 | not detected |  |
| 13 | 18.19 | 19.27 | -1.08 |
| 14 | 27.32 | 27.37 | -0.05 |
| **Median(IQR)** | **27.06 (23.60-27.99)** | **27.16 (24.73-29.08)** |  |
|  | **P=0.047** | |  |

**Table S5 Comparison of the EGFR mutation status between tumor tissue and plasma in EGFR-TKIs naïve patients**

| **EGFR genotype** | **Tissue** | **Plasma (n=20)** | | | |
| --- | --- | --- | --- | --- | --- |
|  |  | **EV DNA** | | **cfDNA** | |
|  |  | **Mutant type** | **Wild type** | **Mutant type** | **Wild type** |
| **Mutant type** | 20 (100.0%) | 11 (55.0%) | 0 | 6 (30.0%) | 0 |
| **Wild type** | 0 | 0 | 9 (45.0%) | 0 | 14 (70.0%) |
| **Sensitivity (%) (95% CI)** | | 55.0% (33.2-76.8) | | 30.0% (9.9 – 50.1) | |
| **Specificity (%) (95% CI)** | | NA | | NA | |

Abbreviations; CI : Confidence interval, NA : not available

**Table S6 Clinical characteristics of patients who developed acquired resistance to 1^st^ or 2^nd^ generation EGFR-TKIs and underwent rebiopsy.**

|  | **Sex** | **Age** | **Primary tumor biopsy** | **Rebiopsy** | | | | | | **EV DNA** | **cfDNA** | **EMSI** | **Response to EMSI** |
| --- | --- | --- | --- | --- | --- | --- | --- | --- | --- | --- | --- | --- | --- |
|  |  |  |  | **Location** | **Size (mm)** | **Method** | **Pathology** | **Result** | **EGFR genotype** |  |  |  |  |
| 1 | F | 54 | E 19 del | Liver | 40 | PCNB | adenoca | success | E 19 del, p.T790M | E 19 del, p.T790M | E 19 del, p.T790M | Olmutinib | PR |
| 2 | F | 57 | E 19 del | RUL | 27 | PCNB | no tumor | failure | NA | E 19 del, p.T790M | E 19 del | Osimertinib | PR |
| 3 | M | 76 | E 19 del | RML | 24 | PCNB | adenoca | success | E 19 del | E 19 del, p.T790M | E 19 del |  |  |
| 4 | M | 64 | E 19 del | RML | 50 | PCNB | suggestive of adenoca | success | E 19 del | E 19 del | WT |  |  |
| 5 | M | 67 | E 19 del | LN | 33 | EBUS | adenoca | success | E 19 del, p.T790M | E 19 del, p.T790M | E 19 del, p.T790M | Olmutinib | PR |
| 6 | F | 83 | p.L858R | RML | 28 | PCNB | adenoca | success | p.L858R | p.L858R | p.L858R |  |  |
| 7 | F | 75 | E 19 del | Lingula | 27 | PCNB | no tumor | failure | NA | 19 del | 19 del |  |  |
| 8 | F | 81 | E 19 del | LUL | 37 | PCNB | no tumor | failure | NA | E 19 del, p.T790M | E 19 del, p.T790M | Osimertinib | PR |
| 9 | F | 70 | E 19 del | RLL | 30 | FOB | adenoca | success | E 19 del | E 19 del | WT |  |  |

Abbreviations; E19 del: exon 19 deletion, WT : wild type, RUL : right upper lobe, RML : right middle lobe, RLL : right lower lobe, LUL : left upper lobe, LN : lymph node, PCNB : percutaneous needle biopsy, EBUS : endobronchial ultrasound, FOB : fiberoptic bronchoscope, adenoca : adenocarcinoma, NA : not available, EMSI : epidermal growth factor receptor mutant selective inhibitor, PR : partial response

**
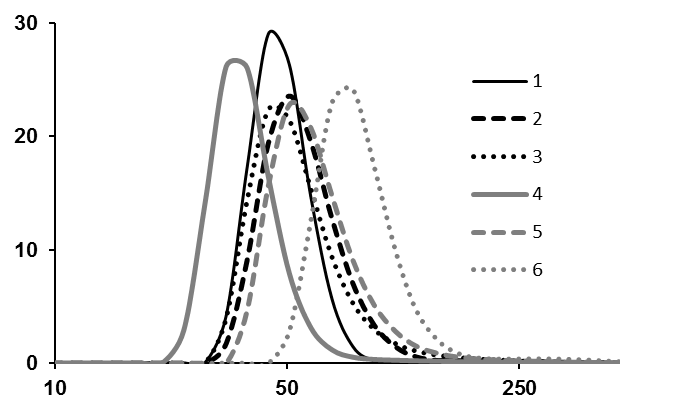
**

**Figure S1 Sizes of purified BALF EVs (n=6)**

**Figure S2 Sizes of purified plasma EVs (n=3)**


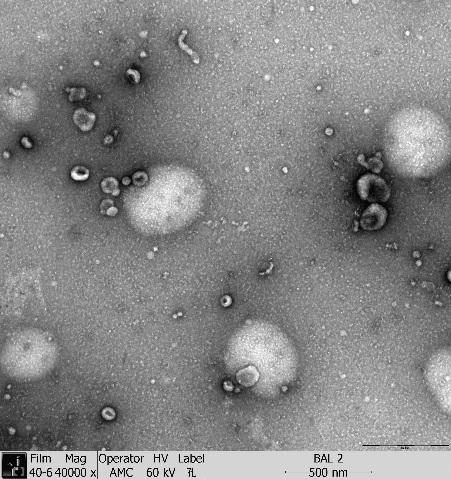


**Figure S3 EM image of BALF EVs**


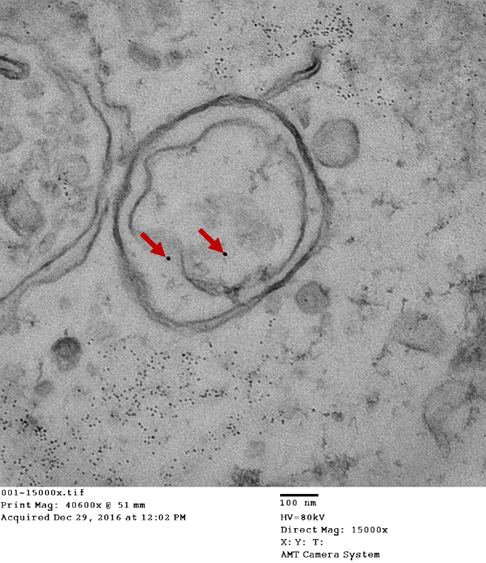


**Figure S4 Immuno-EM images show detection of dsDNA in BALF EVs. Red arrows indicate gold particles.**


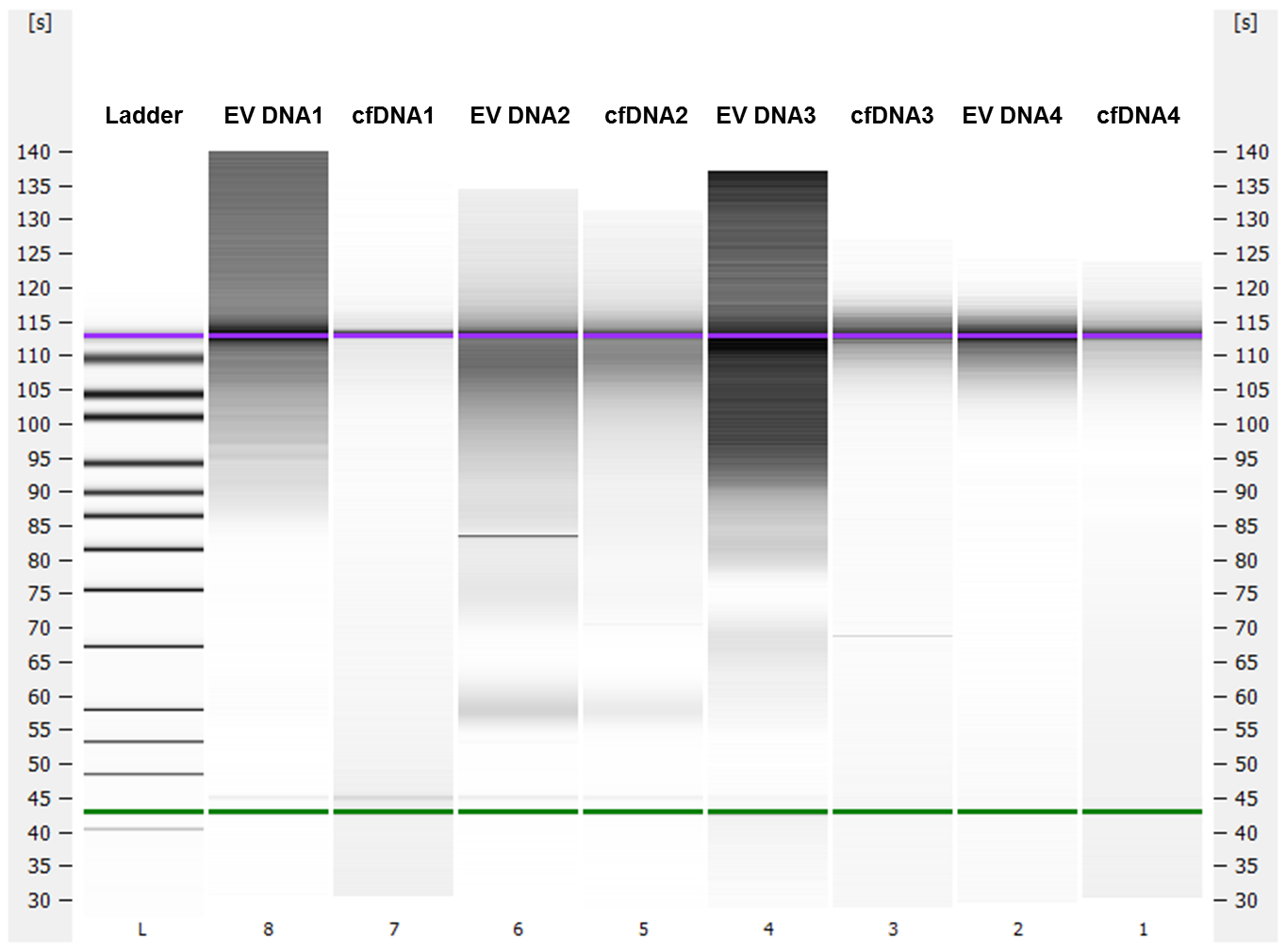


**Figure S5 Gel-like images show the size and amount of EV DNA and cfDNA determined using the bioanalyzer. (n=4)**
